# Charting the Course: Towards a Comprehensive Newborn Screening Program in India

**DOI:** 10.3390/ijns10030043

**Published:** 2024-06-24

**Authors:** Seema Kapoor, Amit Kumar Gupta, B. K. Thelma

**Affiliations:** 1 Genetic & Metabolic Lab, Division of Genetics, Department of Pediatrics, Lok Nayak Hospital & Maulana Azad Medical College, New Delhi 110002, India; 4dramitgupta@gmail.com; 2 Department of Genetics, University of Delhi South Campus, New Delhi 110021, India

**Keywords:** newborn screening program, inborn errors of metabolism, public health, low- and middle-income countries, Delhi State

## Abstract

Integrating health interventions in a growing economy like India, with a birth cohort of 27 million/year, one-fifth of all childbirths, and approximately one-third of neonatal deaths globally, is a challenge. While mortality statistics are vital, intact survival and early preventive healthcare, such as newborn screening (NBS), are paramount. The appalling lack of information about the precise burden of metabolic errors at the state/national level or a mandated program encouraged a feasibility study of NBS in a prospective newborn cohort recruited in Delhi State (November 2014–April 2017) using a public–private partnership mode. The major determinants for effective implementation of universal NBS at the national level and limitations encountered are discussed in this report. Data to generate the ‘core’ panel for screening, sustained training of healthcare personnel, dissemination of the power of NBS to ensure neonatal/societal health to the public, and a ‘national policy’ emerge as priorities in a developing country.

## 1. Background

Integrating health interventions for preventable non-communicable diseases in a growing economy is a challenge. India has 17.78% of the global population with 1,439,803,672 individuals, one-fifth of global childbirths, and one-third of all neonatal deaths [1,2]. The significant decline in infant mortality rates in the recent past [3] underscores the necessity of establishing and adapting a robust national newborn screening (NBS) program, especially as the IMR begins to approach single-digit figures [4], which aligns with WHO guidelines [5] advocating for an NBS program. Despite reaching this figure in some states of India, such as Goa, Kerala, Manipur, and Puducherry, NBS is currently not considered a priority nationwide. However, it is important to note that implementing a comprehensive NBS program could significantly contribute to achieving the goals outlined in the 2014 India Newborn Action Plan (INAP), particularly the target of reducing neonatal mortality to single-digit figures by 2030 [6].

Of the few successful pilot NBS programs in this ethnically, genetically, and geo-graphically distinct yet diverse country, experiences from Chandigarh and Kerala are encouraging [6,7]. An early initiative in Goa elucidated the prevalence of IEMs, but treatment and follow-up arms were neglected; however, with corrective measures, the screening has been reinitiated [8]. No consensus has been reached at the national level for implementation of NBS, although it is being increasingly realized that within the medical dogma, the cure- or treatment-focused reactive approach instead of proactive consideration of prediction and prevention is economically wasteful. Considerations of unquantifiable social and emotional burden to the family, society, and the state are not even discussed.

With this background and the increasing realization of the appalling lack of public health policy for NBS for metabolic disorders, the Science and Engineering Research Board (SERB) of the Department of Science and Technology of the Government of India facilitated the first-ever major initiative to carry out a feasibility study of NBS in Delhi State. Five common and relatively resource-economically treatable disorders, i.e., congenital hypothyroidism (CH), congenital adrenal hyperplasia (CAH), Biotinidase deficiency, Galactosemia, and Glucose 6 phosphate dehydrogenase (G6PD) deficiency [9,10], were selected for screening, consistent with the recommendations of Hinton et al. [11,12].

The extensive experience of the authors in efforts to establish newborn screening in India prior to and during this feasibility study led to identifying contemporary considerations and limitations for implementation of a robust screening program. This article summarizes issues in operational research and translation of a public health program (NBS) as an amalgamation into routine neonatal healthcare and the healthcare infrastructure.

## 2. Methodology

This prospective cohort analysis was conducted in Delhi, India to assess the feasibility of newborn screening (NBS) for selected metabolic disorders and its possible integration into public health policy.

A cohort of 203,400 newborns was recruited from 20 public and private hospitals across Delhi State over 30 months, from November 2014 to April 2017. Five disorders, as mentioned above, were chosen.

Utilizing a public–private partnership model, dried blood spot samples were collected from each newborn and screened for the selected disorders using appropriate biochemical and immunoassay techniques.

Incidence rates of selected disorders, follow-up compliance, and treatment outcomes were documented. Data analysis aimed to assess the effectiveness of the NBS program and its scalability to a national level.

Institutional review board approval was obtained from participating hospitals, and informed consent was secured from parents or legal guardians of all participating newborns.

## 3. Considerations and Limitations

### 3.1. National Planning

The purpose of NBS is to identify the ‘at-risk neonate,’ which is often clinically indiscernible in a pre-symptomatic phase wherein prompt initiation of monitoring and treatment provides the best possible outcomes. Each country or each state, depending upon where the responsibility of ‘health’ as a subject rests, has to decide upon a ‘core panel’ or ‘recommended panel’ of disorders for screening, depending upon the significant impact of the condition(s) on child health and development, incidence of the disease(s) in the screened population, availability and cost of subsequent treatment, the frequency of monitoring required, and the availability of surveillance to assess the optimal impact on child health and development. This, in turn, depends on the feasibility of executing the steps involved in an NBS program, from sample collection to delivery, analysis, reporting, recall and referral, diagnosis confirmation, and follow-up of confirmed positives [13], together with the involvement of pediatric endocrinologists, metabolic physicians, geneticists, and the services of biochemical genetic laboratories. The extent to which these multiple determinants evolve to deliver a comprehensive newborn screening system is driven by the availability of financial resources; testing and reporting expertise; clarity of responsibility; presence of competing priorities; and likely changes in child health quality measures. The most notable limitation currently is the absence of an active national body constituted by all stakeholders at a single platform to discuss a road map in the context of the complex socio-demographic, ethnic, religious, and genetic fabric of India.

### 3.2. Implementation of Screening

The rationale for prioritizing CH lies in the availability of effective and cost-efficient treatment and the feasibility of screening. As the healthcare infrastructure continues to develop, it may become more feasible to expand the NBS program to include other disorders, like sickle cell disease (SCD) and various inborn errors of metabolism (IEMs). Rather than immediately focusing on high technical expertise and potent LC-MS/MS technology, integrating and building a network for CH in the country seems appropriate. G6PD could be considered, as the costs of screening for this disorder (mainly around initial testing and confirmation) may be justified, as its high incidence, the straightforward preventive strategy for its management, and the evidence supporting the effectiveness of screening in reducing clinical issues make it an appropriate choice for NBS programs and long-term savings to the community/population screened. Knowledge of the fact that hemolysis could complicate neonatal hyperbilirubinemia but also accompany the presentation of enteric or dengue fevers amongst other triggers is vital [14,15]. After the core panel is well-established and when the country’s economy as well as its health priorities permit, providing infrastructure and technical expertise for the evaluation and implementation of expanded screening could possibly be considered. Understandably, integration of complex technologies like MS/MS will ensue once the primary fabric is tensile.

Even if NBS has a separate budget allocation at the level of the National Health Mission, its supplementation by other sources, such as Janani Shishu Suraksha Karyakaram (JSSK) and a unique ID with digital health mission integration, is desired to carry out all of the tailored tests in this paradigm if sequential follow-up is required in a subset of newborns identified with a disease [16,17]. Specialty services, such as pediatric endocrinology to supplement NBS labs, have already been partially alluded to. Also, the centers of excellence for rare diseases under the National Rare Disease policy [18] can be considered regional centers for the states they are located in. The UMMID program of the Department of Biotechnology of the Government of India has envisioned the incorporation of NBS as a measure to improve performance indicators of morbidity and mortality in infancy [19]. In the next phase, after implementation of expanded screening at multiple regional laboratories in the country, establishing a QA center in India would be important.

### 3.3. Integration into Existing Healthcare

There has recently been a paradigm shift towards institutional deliveries, which are now 74–88% [20,21]. Overall, the length of stay (LOS) after childbirth is 3.4 days, with 2.1 days for vaginal deliveries and 8.6 days for caesarean section (CS) deliveries. For vaginal birth in public hospitals, one-fourth of the women are discharged with insufficient LOS (<24 h) compared to only 19.2% women in private hospitals [22]. The public-sector-paid births are usually immunized, and samples for NBS are taken at discharge.

States with poor indicators, including absence of skilled birth attendants and delayed first visit of ASHA worker or ANM, perform poorly, and stratified and more incentivized programs are assessing better determinants, which may translate to the fact that training this core set may improve coverage in neonates discharged early as regards to NBS [23].

As recruitment of large numbers of newborns would be central to the success of this initiative, nurses, midwives, and Accredited Social Health Activists [ASHAs] [24] would be effective entry points in the program. A set module with training of trainers if initiated at a national level and conducted on a routine basis to update new staff recruits as a continuing medical educational initiative is desirable. Developing training materials for NBS requires a collaborative effort among healthcare professionals, public health authorities, and educational institutions. Training sessions and workshops will effectively disseminate the materials, with regular assessments ensuring adherence to standardized procedures. A medical education initiative engages stakeholders, such as government health departments, medical associations, and academic institutions.

### 3.4. Community Awareness

In the absence of family history and in cases of perceived normal appearance of the newborn, it is extremely hard to explain NBS in an illiterate/semi-literate Indian context. Constant large-scale efforts to educate the general public regarding IEMs and the powerful screening/diagnostic tools and effective therapeutic interventions available are imminent. Creating a brochure or website with information on this group of disorders, including the need for testing, frequency of follow-ups, availability of specialty services, designated public centers, and optimal time frames, would be beneficial. However, considering India’s literacy rate is only 74.37% (an increase of 5.07% from 2011) [25], a different strategy is needed. This might include using visual aids, community health workers, and mobile health initiatives to reach a broader audience and ensure effective communication. Involvement of a media celebrity, as with the successful oral polio vaccine initiative, advertisements, as designed for thalassemia and tobacco use prevention, creation of a movie or an educational documentary, and mass media programs on television/radio are a few suggested measures. Involvement of volunteers from high school and college students was seen to be effective in the Philippines [23]. Of note, metropolitan cities in India now have effective mobile phone usage, and around 34 million smartphone units were shipped across India in the second quarter of 2021. With a penetration rate of 54% in 2020 and estimated to reach 96% by 2040 [24], limitations of recall may considerably decrease. An informative audio clip along the lines of those for COVID-19 in recent times may be a repetitive endorsement of this program to mobile-phone-enabled citizens. Nukkad-nataks, popularly known as street shows, are particularly useful for mass communication across all literacy levels. Engaging community health mobilizers and workers may help in allaying anxiety and promoting greater acceptance at the community level.

### 3.5. Costs to the Society

Assessing the costs and benefits of implementing a national newborn screening policy in India necessitates a comprehensive approach by weighing immediate financial implications against long-term societal gains. Research indicates that the financial burden of preventable diseases far surpasses the expenditure on screening, underscoring the cost-effectiveness of investing in NBS programs over time [26]. For instance, a study revealed that the cost of a single screening test for CH using enzyme-linked immunosorbent assay (ELISA) amounts to just USD 5.00, a nominal expense in comparison to the potential lifelong costs associated with untreated CH [27].

Determination of the cost–benefit ratio is a complex issue because of the intricacies in judgmental differences. Even if one were to think beyond the confines of ‘health for all,’ judgement regarding simple and easily performed tests appears straightforward. The benefits are both obvious tangible gains to the family and intangible ones to society at large. CH as an identified target has been included in the Rashtriya Baal Swasthya Karyakram (RBSK) in the national program for the nation [27].

### 3.6. Identification of Carrier Status

Screening for some disorders (for example, screening for hemoglobinopathies) has the potential to identify carriers of the disorders with the potential for stigmatization of those carrying the disorders. After much discussion, it was decided that this potential harm from screening outweighed the benefit of early detection and screening for hemoglobin disorders not included in the initial panel. While broad education about carrier status and its implications can mitigate stigma, challenges exist in effectively disseminating this information, particularly in regions with diverse languages and varying levels of information access and literacy. With one of the largest telecom services across the world and the increasing use of social media, broad education of the population about the meaning and implications of carrier status can be provided in every vernacular language, but, given the transitional phase of the penetration of internet-based knowledge gathering and literacy levels in the Indian population, this will be difficult.

### 3.7. Sample Transport

Early detection of metabolic disease requires all steps of the screening pathway (including transport of samples to the laboratory) to be carried out in the most timely way possible.

Efficient and timely transport of samples is essential for early detection of metabolic diseases. Due to logistical challenges, hand-held transport by designated project personnel was adopted for transferring lancets and filter papers to collection centers and receiving sample cards from hospitals in Delhi. Issues like improper collection and transportation can compromise the quality of dried blood spot specimens, necessitating standardized procedures across all collection sites. While certain options, such as courier services and postal services, offer reliability and cost-effectiveness, dedicated transport networks ensure sample integrity at the expense of significant investment. While using the Indian Post for sample transport from remote rural settings is a potential solution, the decline of postal services internationally makes courier services a more viable alternative for timely deliveries. Balancing cost and sample quality is paramount for the success of NBS programs. Issues, such as improper collection, specimen contamination, in-complete drying, and transporting at an inappropriate temperature, can alter the quality of the DBS specimen [28]. Standardizing these procedures across all collection sites is crucial for ensuring the reliability of NBS results [28]. Efficient transport methods, such as courier services and postal services, offer reliability and cost-effectiveness, while options like dedicated transport networks ensure sample integrity but require significant investment. Balancing cost and sample quality is crucial for successful NBS programs.

### 3.8. Follow-Up

Positive NBS tests were communicated to pediatricians through email, SMS messages, and calls on landlines and cell phones. PDF reports were emailed, but the reviewing physicians found it burdensome to go through all of the reports, and even urgent prompts were not recognized or put aside for later reading, so color-coding was introduced, with urgent prompts in red and semi-urgent in yellow. Tracking down the presumptive affected infants and those requiring second samples to complete screening was the most difficult aspect of the study. A lack of standard operating procedures, early hospital discharges, and unreliable contact details were other common problems. Most of the centers in Delhi cater to migrant populations who relocate to their homes soon after an uneventful delivery. The mobile phone may be switched off, or the number may be changed (frequent changes of plan are common due to new incentives offered by the service providers).

A particular issue has been parental refusal of follow-up testing. This may be due to inadequate pre-test counselling (e.g., including the causes of screened disorders to minimize parental self-blaming), but the number of daily deliveries (20–80 in some centers) made one-to-one counselling impractical. A voluntary ‘opt out’ philosophy may be a practical way of implementing NBS in a developing country with high birth rates across many public health facilities. Pre-test counselling using multiple modalities to provide broad information about screening is highly desirable. Extended family as an important target to counsel may be a valuable addition in the context of an Indian family setting.

### 3.9. Premature and Sick Newborns

Sample collection from these infants is a challenge. Pediatricians may not consider NBS as important as other medical interventions, although the results may indicate the cause of symptoms, such as hypoglycaemia. Creating awareness among physicians and paramedical staff is a critical requirement for success in these infants. Many inherited metabolic disorders (e.g., Galactosemia, fatty acid oxidation defects, organic acidemias) as well as CAH present with hypoglycaemia and can be easily detected pre-symptomatically through NBS [29]. NBS may thus be very useful for a treating pediatrician/neonatologist. Imparting knowledge of NBS at undergraduate and postgraduate levels at medical schools may be a long-term solution.

### 3.10. Prosaic Interests and Potential Harms

Finally, along with realizing the long-term benefits of currently screening the common disorders, such as CH and G6PD deficiency, the potential for inadvertent harms has to be tackled. These include promotion by certain private companies engaged in manufacturing of test reagents or dietary products. Though the Diet for Life initiative [30] has encouraged in-house manufacturing of special diets for medical purposes, a venture from a few Indian pharmaceutical companies for affordable diets and emergency drugs for IEMs in the country is imperative for a successful launch of expanded NBS, and this is currently being witnessed.

## 4. Summary

This narrative is by no means a complete list of factors and concerns in the successful planning and roll-out of a program in an LMIC. Developing support for implementation of NBS requires three arms of education to ensure neonatal/societal health and reduce genetic disease burden. Within the health sector, awareness of the value of screening must be developed among healthcare professionals in pediatrics and maternity care from the time screening is planned and then included in sustained training, and, importantly, among healthcare funders (both public and private). In the community, increased knowledge of the impact screening can have on the health of babies, the importance of follow-up, and basic human genetics would favorably impact participation and follow-up. Selection of conditions for the screening panel must be informed by local needs, especially in a large country with ethnically distinct and diverse regions, and this may require region/state-wise epidemiological studies.

Within public funding bodies, issues of competing priorities, lack of personal experience, and under-recognition of the late repercussions of morbidity as a state/national responsibility seem to be barriers to introducing a law to mandate screening.

Finally, implementation must consider both local healthcare systems and infrastructure so that samples can be collected most efficiently and transported to the laboratory in a timely manner.

## Data Availability

Data will be made available upon request to interested researchers.
